# The role of intra-articular administration of Fetuin-A in post-traumatic knee osteoarthritis: an experimental study in a rat model

**DOI:** 10.1186/s40634-019-0194-4

**Published:** 2019-06-17

**Authors:** Eleni Pappa, Savvas Papadopoulos, Laskarina-Maria Korou, Despina N. Perrea, Spiridon Pneumaticos, Vasileios S. Nikolaou

**Affiliations:** 1 “KAT” General Hospital of Athens, Nikis 2, 14561 Kifisia, Greece; 2 Department of Pathology, “Hygeia” General Hospital of Athens, Athens, Greece; 3 Laboratory of Experimental Surgery and Research “N.S. Christeas”, Athens Medical School, Athens, Greece; 4 3rd Department of Orthopaedics, KAT Hospital, National and Kapodistrian University of Athens, School of Medicine, Athens, Greece; 5 2nd Department of Orthopaedics, Agia Olga Hospital, National and Kapodistrian University of Athens, School of Medicine, Athens, Greece

**Keywords:** Fetuin-a, Rats, Osteoarthritis, Knee, Fetuin-A

## Abstract

**Background:**

The purpose of this study is to investigate the possible attenuating role of the intra-articular administration of Fetuin-A in post-traumatic secondary osteoarthritis in rats, and also its effect on the systematic levels of interleukins (ILs)-2,4,7, bone morphogenetic proteins (BMPs) 2, 4, 7, C-Reactive Protein (CRP) and Fetuin-A.

**Methods:**

Thirty male Sprague Dawley rats were separated in two groups where post-traumatic osteoarthritis was induced surgically by Anterior Cruciate Ligament Transection and the transection of the Medial Collateral Ligament of the right knee. In the Control Group, only the surgical intervention took place. In Fetuin Group, along with the induction of osteoarthritis, a single dose of bovine fetuin was administrated intra-articularly, intra-operatively. Both groups were examined for 8 weeks. The levels of interleukins, bone morphogenetic proteins, Fetuin-A and C-Reactive Protein were evaluated by ELISA of peripheral blood in three time periods: preoperatively, 5 and 8 weeks post-operatively. Osteoarthritic lesions of the knee were classified according to the Osteoarthritis Research Society International Grading System and the Modified Mankin Score, by histologic examination.

**Results:**

IL-2 levels were significantly decreased in the Fetuin Group. No statistical difference was signed on the levels of IL-7, BMP-2,4,7 and Fetuin-A between the two groups. CRP levels were significantly increased in the Fetuin Group in 5 weeks of the experiment. Fetuin Group signed better scores according to the OARSI classification system and Modified Mankin Score, without any statistical significance.

**Conclusions:**

Intra-articular administration of Fetuin-A restrictively affected the progression of post-traumatic arthritis in rats, as only the levels of IL-2 were decreased as well as limited osteoarthritic lesions were observed on the Fetuin Group.

## Background

Osteoarthritis (OA) is a musculoskeletal condition where not only the deterioration of the articular cartilage is present but also a gradual subchondral erosion of the bone exists (Glasson et al. 2007). It is well described that the joint arthritis consists of an extended inflammatory response which is introduced by several cytokines, which are produced by the local action of macrophages, such as interleukins and matrix metalleinoproteinases (MMPs). The cytokines above are present in serum and synovial fluid and all have the potential to lead to the local cartilage decrease (Attur et al. 2013, Huch et al. 1997). Additionally, collagen type 1 is restructured to collagen type 2, while the joint chondrocytes are declined too. It is widely known that Interleukin (IL-1) leads the inflammatory process of arthritis, so that MMPs are produced, and as a result the cartilage relegation takes place. Acute phase proteins (APPs), including C-reactive protein (CRP), are elevated in patients’ serum with severe stages of osteoarthritis (Wang et al. 1997).

The protein Fetuin-A, which is a member of APPs, has recently drew attention concerning its potential role in attenuating the inflammatory response (Pedersen 1944, Wang et al. 2010). Current literature has described a complete structure of the oligosaccharides of Fetuin-A, where mannose and N-acetyl-glycosamine of a ratio of 3:3:3:5 are present (Baenziger and Fiete 1979; McNulty et al. 2011). Fetuin-A was first declared as a fetus major plasma protein and is also described as alpha-2-HS-glycoprotein (AHSG). It is expressed in the liver, kidney, gastrointestinal tract, skin and brain, during the fetal growth (Pappa et al. 2017). Recently, Fetuin-A drew interest regarding its possible protective role against inflammation in injury or infection, being classified as a positive APP thanks to the mediation of High Mobility Group protein (HMGB1), but also as a negative APP because of a regulation of pro-inflammatory cytokines such as tumor necrosis factor (TNF), interleukins (IL) -1, IL-6, IL-12, IL-18 and interferon (IFN)-γ (Sluijs et al. 1992; Kraus et al. 2011). In 1998, *Wang* et al proved, through examination of murine cell cultures, that Fetuin-A can be utilised by the local macrophages as an opsonin for macrophage deactivating molecules. Fetuin is used by macrophages to assess the abundance of extracellular spermine, which, moreover, downregulates synthesis of proinflammatory cytokines and inhibits excessive inflammation on the local tissue (Sturmer et al. 2004). In addition, *Wang* et al in 2010 proved the protective role of Fetuin-A in the ischemic cerebral inflammation in rat models together with the suppression of sepsis mediators in late stages of sepsis in the same animal model of rats (Wang and Sama 2012).

Regarding the anti-inflammatory potential of Fetuin-A and its ability in regulating the pre-inflammatory cytokines, we hypothesized that the local intra-articular administration of Fetuin-A in a Post-Traumatic Osteoarthritis (PTOA) rat model could inhibit the progress of post-traumatic arthritis of the knee joint, but also to modify the systematic levels of IL-2,4, 7, BMP-2,4,7, Fetuin-A and CRP.

Concerning the role of interleukins, firstly IL-2 secretion has been studied in osteoarthritic populations, and is being characterized as elevated in the first stages of the disease (Wang et al. 1997). Moreover, Interleukin (IL)-7 is a cytokine involved in the regulation of B cell development and survival. In arthritis, IL-7 might play an important role since its levels are reported elevated in the synovial fluid and serum from rheumatoid arthritis (RA) patients, in comparison with osteoarthritis (OA) patients. Also,mice treated with IL-7 during collagen type II-induced arthritis (CIA) showed an expansion of the B and T cell pool and increased joint destruction (Ponchel et al. 2015).

Regarding BMPs, BMP-7 is crucial for the maintenance of homeostasis in articular cartilage, as it emerges in the superficial layer of articular cartilage concomitant with the expression of BMP receptors (BMPR-IA, IB, and II) and contributes to the re-expression of the chondrocyte phenotype of dedifferentiated cells. Also, it increases the synthesis of tissue inhibitor of metalloproteinase (TIMP), but also enhances the expression of Insulin Growth Factor I (IGFI), and chondrocyte cytoskeletal proteins (Chubinskaya et al. 2007). Nevertheless, BMP-2 seems increased in OA, accordingly to the disease severity. In severely damaged cartilage, cellular localization of BMP-2 production extends to the deep zone. It is also suggested that chondrocytes depend on the cartilage remodeling and repair functions of BMP-2 to maintain anabolic metabolism during the progress of OA, raising the possibility that BMP-2 leads a critical role in the development of knee OA (Liu et al. 2015, Little and Hunter 2013). Additionally, BMP-4 stimulates the production of extracellular matrix in chondrocytes and supports the healing of bone fractures. Overexpression of BMP-4 leads to increased cartilage formation and chondrocyte differentiation (Bramlage et al. 2006).

Animal models are frequently used in experimental research on osteoarthritis (Chambers et al. 1997, Lampropoulou-Adamidou et al. 2014). In the specific study, secondary post-traumatic osteoarthritis (PTOA) of the knee joint was examined. In concerns of animal models, PTOA is the most commonly studied, especially via invasive experimental models of osteoarthritis (Lampropoulou-Adamidou et al. 2014). The surgical induction of osteoarthritis in rats is highly reproducible and consists a legit choice for short term studies, while multiple studies in the present literature exist and materials are also easily available. Since studies have shown that the invasive surgical protocols of PTOA induction in rat models are accompanied by the certain advantages above, we considered the most suitable animal model for the specific study could be the rat. Furthermore, the Anterior Cruciate Ligament Transection (ACLT) together with the transection of the medial collateral ligament of the knee (MCLT) was chosen as the surgical protocol for PTOA induction (Kuyinu et al. 2016; Goldring and Goldring 2006)

To the best of our knowledge, the effect of the intra-articular administration of Fetuin-A on the progress of secondary osteoarthritis has not been investigated to date. If our medical hypothesis could be confirmed, then a new agent could be added in the prevention of the rapid progress of knee osteoarthritis and the role of intra-articular administrated anti-inflammatory drugs in secondary osteoarthritis could be examined from a different view.

## Methods

### Animal models and experimental design

Thirty male, 6 months old, Sprague Dawley rats were housed per 3 animals in stainless steel wire-bottom cages. The rats were kept in a temperature-controlled environment (19 ± 1 °C with 50 ± 5% relative humidity) with a 12-h light/dark cycle (5:30 am to 5:30 pm) in an air-conditioned room with 15 air changes/h and had free access to food and tap water. During their stay, the everyday consumption of tap water was measured individually. The animals were examined clinically by a veterinarian throughout the entire experimental period. All possible steps were taken to avoid animal suffering at each stage of the experiment. The interventions that could cause discomfort or stress to the animals (ie blood samplings) were performed under mild sedation which was achieved by xylazine-ketamine intra-muscularly (I.M). The use and treatment of the rats was in accordance with the European Communities Council Directive of September 22, 2010 (276/33/20.10.2010) and the protocol was approved by the competent Veterinary Directorate of Athens Prefecture, Greece (Approval No.: 908/23.02.2016).

Animals were allowed 2 weeks of acclimatization after arrival and then were randomly assigned (sealed envelope) in two experimental groups. Animals in the Control group (*n* = 15) were operated in the right knee where Anterior Crucial Ligament (ACL) + Medial Collateral Ligament (MCL) tear were performed, and animals in the Fetuin Group (*n* = 15) were operated in the right knee where the same surgical procedure was performed along with the administration of 1 μl of 3% solution of bovine fetuin in normal-saline, intra-articularly, with the use of ultra-fine needle insulin syringe, intraoperatively. The administration was made with precision by entering in the intra-articular space through the anterolateral joint line, with the knee flexed, directing the needle towards to intercondylar notch. There was not any leak of the fluid noticed. Also, the limited bleeding during the procedure did not inhibit the administration of the fetuin solution. The bovine fetuin was in the form of lyophilized powder and was commercially available to the laboratory. The control group did not receive the solution of bovine fetuin. Regarding the surgical procedure, the medial meniscotibial ligament (MMTL) anchors the distal medial meniscus (DMM) to the tibial plateau, while the anterior cruciate ligament (ACL) restricts the tibia from moving anteriorly, relative to the femur. The surgical approach for both ACL and MCL transection was made with a 5 mm longitudinal incision over the distal patella to proximal tibial plateau. The joint capsule immediately medial to the patellar tendon was incised with a # 11 blade and the joint capsule was opened with micro-iris scissors. Blunt dissection of the fat pad over the intercondylar area was then performed to expose either the intercondylar region, providing visualization of the anterior cruciate ligament or the meniscotibial ligament of the medial meniscus. Mild hemorrhage from the fat pad upon blunt dissection was controlled by pressure from absorption spears.

The ACL is lateral to the posterior cruciate ligament (PCL), which was only rarely visualized in our surgical approach, in the posterio-medial intercondylar region. For the ACL transection, the patella was dislocated medially to give greater exposure of the femoraletibial joint. When the patella was dislocated, the cartilage was kept moist with saline as required. The ACL was transected with a micro-surgical knife under direct visualization, avoiding the PCL, and complete transection confirmed by the presence of anterior drawer. The medial meniscotibial ligament (MMTL) anchors the DMM to the tibial plateau. The fat pad over the cranial horn of the medial meniscus was dissected with Jewelers forceps. The MMTL was identified running from the cranial horn of the medial meniscus laterally onto the anterior tibial plateau. Care was taken to identify and avoid the lateral meniscotibial ligament (LMTL), which is posterior and has fibers running in a similar direction. Sectioning of medial meniscotibial ligament with micro-surgical scissors, micro-surgical knife or # 11 blade, with the blade directed proximolaterally, gave destabilization of the medial meniscus. With the DMM intact, there is a greater congruency and area of contact between the articulating structures, providing a larger region to transmit the weight-bearing forces. As a result, medial displacement of the DMM occurs, and weight bearing is focused across a smaller area, leading to increased local mechanical stress. Since the rat knee is flexed during weight bearing, this leads to greater stress on the posterior femur and central tibia, predominantly on the medial side. The joint capsule was closed with a continuous 2–0 tapered Vicryl suture and the subcutaneous layer with 2–0 cutting Vicryl suture. The skin was closed by the application of tissue adhesive. Rats had excellent mobility within 2 h after either surgery.

The practice of the 3Rs (Replacement, Refinement, Reduction) was adhered to. The control group underwent the least of the interventions. On the other hand, the results of the Fetuin Group are anticipated to a greater extent, as shown in previous researches. The examination period was 8 weeks, as proposed by the literature (Baenziger and Fiete 1979; Bendele 2001; Gerwin et al. 2010).Also, Fetuin-A was administered in the form of bovine fetuin. We decided to administer bovine fetuin through intra-articular injections, on the grounds that the animals were provided with mild sedation so that to eliminate the stress which could be caused.

Blood samples were collected at 0 (T0), 5 (T1) and 8 (T2) weeks of the surgical intervention in the right knee for the PTOA induction in both groups. After the blood collection in each time period, the animals were euthanatized by administration of ether nebulizer preceded by Intra Muscular (IM) ketamine- xylazine sedation at 5 and 8 weeks respectively. Their knee joints were both removed, as the left knee joint was used as control, and their femur-tibia-patella-malleolus bones were excised.

### Blood analyses

#### Biochemical measurements

Peripheral Blood samples were collected for the determination of IL-2, IL-7, bone morphogenetic proteins BMP-7, BMP-4, BMP-2, Fetuin-A, and CRP levels**,** in order to determine not only the local inflammation response but also the systematic inflammation response of the rats.

Blood samples were stored in blood collection microtubes of a capacity of 1 ml. The blood samples were collected by Ependorf tubes through the rats orbital sinus. The samples were stored at -80 °C, in liquid nitrogen freezers until analysis.

#### ELISA

Serum interleukins, BMPs, Fetuin-A and CRP levels were determined by enzyme-linked immunosorbent assay (ELISA) using commercially available kits. The ELISAs on the blood samples were performed in the same time for all the time points. The optical density (OD value) of each factor was determined, using a micro-plate reader (BIORAD 630), to 430 nm. (Rat CRP ELISA Kit, Wuhan Fine Biotech Co,Ltd., Wuhan City, Hubei Province China, Catalog No ER0016, Rat IL-7 ELISA Kit, Wuhan Fine Biotech Co,Ltd.,, Catalog No ER0146, Rat Ahsg ELISA Kit, Wuhan Fine Biotech Co,Ltd., Catalog No ER0246, Rat BMP-4 ELISA Kit, Wuhan Fine Biotech Co,Ltd., Catalog No ER0080, Rat IL-2 ELISA Kit, Wuhan Fine Biotech Co,Ltd., Catalog No ER0039, Rat BMP-7 ELISA Kit, Wuhan Fine Biotech Co,Ltd., Catalog No ER0772, Rat BMP-2 ELISA Kit, Wuhan Fine Biotech Co,Ltd.,

Catalog No ER0010).

#### Tissue analysis

##### Histology

Histologic examination of the knee joint was performed as described from Pritzker et al. (Pritzker et al. 2006). In brief, the isolated knee joint was cleaned from the muscle tissue and was cut longitudinally. The knee isolation took place immediately after the rats euthanization ether at 5 or 8 weeks respectively. Specimens included the femur together with the tibia and malleolus bones. The bony structure as well as the joint connective tissue were then embedded in normal saline gauzes and preserved fresh frozen in -80 °C.Before histologic analysis, the specimens were preserved in 18 °C, embedded in 10% formalin buffer for 24 h. Then they were embedded in Ethylenediaminetetraacetic acid (EDTA) 10% for decalcification for another 24 h, and then embedded in paraffin. Paraffin sections of 3 μm thickness of the knee joints were cut and stained with eosin and hematoxylin for further microscopic examination, in frontal sections of the right knees and both frontal and sagittal sections the left knee too, as a control. No artefacts were observed. An expert, blinded to the intervention groups performed the histologic examination. There are also additional staining procedures for the cartilage degeneration monitoring, such as Safranin O/Fast green, Masson’s Trichrome, Methylene blue, Picro-sirius Red, Toluidine blue or immunohistochemistry, which were not included in our study.

### Image analysis

The evaluation of the knee articular surface was performed using image analysis. Images were digitalized using a light microscope (Olympus BX61, Athens, Greece) with an attached digital camera (Olympus DP70, Athens, Greece). Images were loaded to a computer equipped with the appropriate software (Olympus Micro/DP-BSW, Athens, Greece) and the inflammatory adhesions of the articular surface were measured and compared to the Osteoarthritis Cartilage Histopathology Assessment System (OARSI) Classification System. The OARSI Osteoarthritis Cartilage Histopathology Assessment System is based on histologic features of OA progression (Pritzker 2003). All the grades and stages assume that the tissue reaction observed has microscopic features characteristic of OA activity (Pongratz et al. 2014). The system employs analysis of a standard block/section assessment by grade, stage of arthritis with subsequent calculation of an arthritis score. Each standard block/section for grade/stage assessment is confined to one articular surface and subjacent tissues from one joint compartment, e.g., medial femoral condyle. Microscopic sections are assessed at low power magnification [48]. The main concept underlying the OARSI grading is that whatever the biologic mechanisms, the earliest cartilage changes in osteoarthritis are observed near to the cartilage surface. As cartilage breakdown becomes more severe, increasingly deeper cartilage becomes involved. Ultimately, the cartilage becomes eroded completely and the subjacent bone becomes the articular surface. With normal cartilage as grade 0, osteoarthritis severity is divided into six grades. Grades 1–4 involve articular cartilage changes only, whereas grades 5 and 6 involve subchondral bone too (Pritzker 2003).

Additionally, we used the modified Mankin score as proposed by Sluijs et al. in 1992, which offers a more comprehensive outline of the osteoarthritis staging, and is frequently used in animal experimental models. The Mankin score is a combined score assessing structure (0–6 points), cellular abnormalities (0–3 points), matrix staining (0–4 points), and tidemark integrity (0–1 point). As a result, 0 points implies normal cartilage, whereas 14 points represents the most severe cartilage lesions.

The histopathological gradation of the severity of OA according to Mankin appeared to be directly correlated with the metabolic state of the chondrocytes in the different stages of OA (Mankin et al. 1971, Sluijs et al. 1992).

#### Statistical analysis

For the statistical analysis of these data, we chose to work with non-parametric tests, because of the rather small sample size and the non-normality of the distributions of interest. The statistical analysis included: **a)** paired sample Wilcoxon test **b)** Mann – Whitney U Test. Results were expressed as the mean ± SD. Statistical analysis was performed in IBM SPSS v23 (IBM Corp. Released 2015. IBM SPSS Statistics for Windows, Version 23.0. Armonk, NY: IBM Corp.) A value of *p* < 0.05 was considered statistically significant.

## Results

### Effect of Fetuin-a administration on the body weight of the animals

During the study period, all of the rats gained weight and there was no statistical difference in the mean weight gaining between the groups. The mean body weight of the animals of the control group at 5 and 8 weeks was 571 ± 48 (w ± SD) g and 543 ± 41, respectively. The mean weight of the animals of the fetuin group of the experimental study was 556 ± 37 g and 545 ± 30 g on 5 and 8 weeks respectively. The baseline weight of the control group was 530 ± 35 g and for the fetuin group 531 ± 39 g. These results are presented in Table 1.

**Table 1:** Body weight, IL-2, IL-7, BMP-2,BMP-4, BMP-7, ASHG and CRP in the two groups of animals during the entire experimental period^α^

	GROUP	0 Weeks	5 Weeks	8 Weeks
Body Weight (g)	Control	530 ± 35	571 ± 48	543 ± 41
	Fetuin	531 ± 39	556 ± 37	545 ± 30
IL-2 (OD)	Control	0,12 ± 0,06	0,20 ± 0,04	0,4 ± 0,05
	Fetuin	0,12 ± 0,01	0,23 ± 0,04	0,17 ± 0,03^b^
IL-7 (OD)	Control	0,20 ± 0,05	0,22 ± 0,09	0,25 ± 0,09
	Fetuin	0,16 ± 0,06	0,3 ± 0,16	0,49 ± 0,45
BMP-7 (OD)	Control	0,05 ± 0,008	0,09 ± 0,001	0,11 ± 0,001
	Fetuin	0,05 ± 0,008	0,12 ± 0,009	0,09 ± 0,001
BMP-4(OD)	Control	0,11 ± 0,003	0,21 ± 0,04	0,25 ± 0,04
	Fetuin	0,1 ± 0,003	0,25 ± 0,03	0,22 ± 0,02
BMP-2(OD)	Control	0,18 ± 0,1	0,15 ± 0,11	0,21 ± 0,17
	Fetuin	0,17 ± 0,09	0,24 ± 0,15	0,28 ± 0,12^a^
ASHG (OD)	Control	1 ± 0,07	1,68 ± 0,1	1,61 ± 0,15
	Fetuin	0,98 ± 0,10	1,64 ± 0,26	1,63 ± 0,22
CRP(OD)	Control	0,65 ± 0,14	0,76 ± 0,34	0,95 ± 0,2
	Fetuin	0,68 ± 0,14	0,93 ± 0,12^c^	1,12 ± 0,13^a^

Paired Samples Wilcoxon test adjusted with Benjamini-Hochberg procedure

^α^Data are presented as mean ± SD, ^a^ *p* < 0.05 vs. baseline, ^b^*p* < 0.05 vs. 5 weeks, ^c^*p* < 0.05 vs. Control group, Control Group (*n* = 15), Fetuin Group (*n* = 15)

### Biochemical measurements

**Fig. 1:**
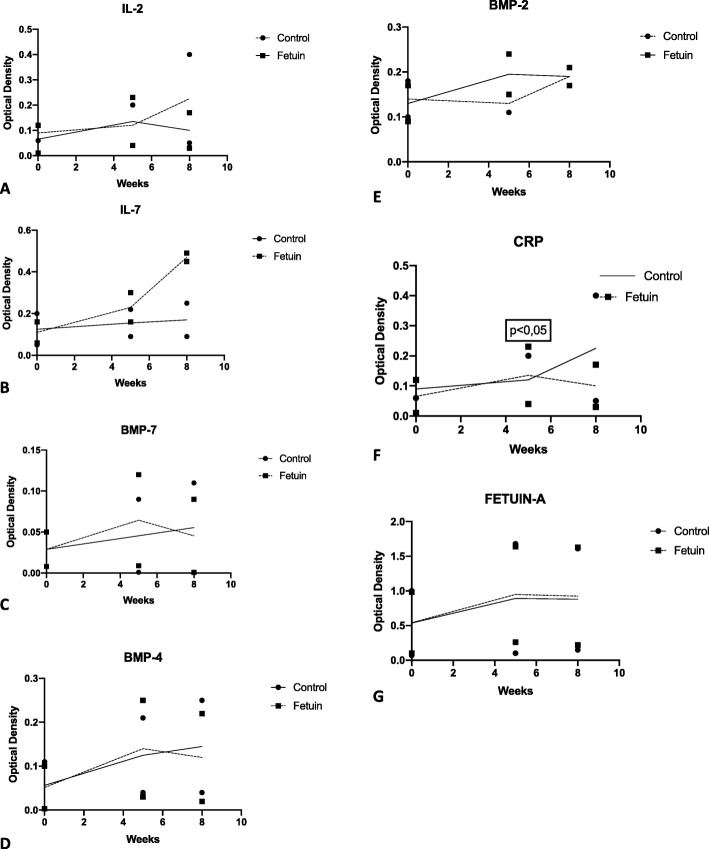
Measure of IL-2, IL-7, BMP-7, BMP-2, BMP-4, CRP and Fetuin-A levels. The levels at 0, 5 and 8 weeks are presented as mean ± SD. **a** The trend of the mean IL-2 levels during the experiment for the control and fetuin group. **b** Mean IL-7 values of the 2 groups during follow up. **c** Mean BMP-7 values of groups during the follow up. **d** Mean BMP-4 values of the 2 groups during the experiment. **e** The trend of the BMP-2 levels during the experiment. **f** The mean CRP values between the two groups during the experiment Statistically signifant difference was noted between the Control and Fetuin Group in 5 weeks of the experimental study. **g** The mean values of Fetuin-A of the control and fetuin group

As mentioned in Table 1 and Fig. 1, the baseline laboratory measurements of all groups were able to be compared at the beginning of the study.

The mean OD of IL-2 of Control Group in 5 and 8 weeks was 0,20 ± 0,04 and 0,4 ± 0,05 respectively, but without statistical difference. In Fetuin Group, the IL-2 levels were increased in 5 weeks (0,23 ± 0,04) but decreased in 8 weeks (0,17 ± 0,03), with a statistical difference in correlation with the control group (*p* = 0.04). There was not any statistical difference in comparison with baseline in both groups. (0,12 ± 0,06 for the control group and 0,12 ± 0,01 for the fetuin group).

IL-7 levels followed and upward trend in 5 and 8 weeks in both groups. In control group, the baseline mean OD of the IL-7 was 0,20 ± 0,05, 0,22 ± 0,09 in 5 weeks of the experiment and 0,25 ± 0,09 in 8 weeks. On the other hand, in Fetuin Group, the mean levels of IL-7 were 0,16 ± 0,06 at the beginning of the experiment, 0,3 ± 0,16 on 5 weeks and 0,49 ± 0,45 on 8 weeks. There was not any statistical difference detected between the groups.

BMP-7 levels seemed increased in control group, as the mean baseline levels were 0,05 ± 0,008, on 5 weeks 0,09 ± 0,001 and on 8 weeks 0,11 ± 0,001, without any statistical difference. In fetuin group, BMP-7 levels increased in 5 weeks (0,12 ± 0,009 from the baseline levels 0,05 ± 0,008), but in 8 weeks they signed a downward trend (0,09 ± 0,001) without any statistical difference (*p* > 0.05). No baseline statistical difference was noticed.

BMP-4 signed increased levels in Control group (baseline 0,11 ± 0,003, in 5 weeks 0,21 ± 0,04 and in 8 weeks 0,25 ± 0,04). In Fetuin group, BMP-4 levels seemed increased in 5 weeks (0,25 ± 0,03) but then declined in 8 weeks (0,22 ± 0,02), without any statistical difference. Baseline statistical difference was absent. (0,11 ± 0,003 for the control group and 0,1 ± 0,003 for the fetuin group).

BMP-2 in Control group was 0,15 ± 0,11 in 5 weeks and 0,21 ± 0,17 on 8 weeks without any statistical difference. In Fetuin group, BMP-2 in 5 weeks was 0,24 ± 0,15 and in 8 weeks 0,28 ± 0,12, which increased in a statistically significant way in correlation with baseline levels (*p* = 0.045).

Fetuin-A levels in Control group in 5 weeks were 1,68 ± 0,1 and then decreased to 1,61 ± 0,15 at 8 weeks, without any statistical difference. In Fetuin Group, Fetuin-A levels were 1,64 ± 0,26 in 5 weeks and 1,63 ± 0,22 in 8 weeks. There was not any statistical difference observed between groups and baseline levels.

Mean CRP levels seemed increased in Control Group, in 5 and 8 weeks (0,76 ± 0,34 and 0,95 ± 0,2 respectively) without statistical difference. On the other hand, in Fetuin Group, the levels of CRP in 5 weeks were more increased in a statistical significant way in comparison with Control Group (0,93 ± 0,12, *p* = 0.043), and in 8 weeks, the CRP levels in Fetuin Group were more increased in comparison with the baseline levels with a statistically significant difference. (1,12 ± 0,13, *p* < 0.05).

### Histology

**Fig. 2:**
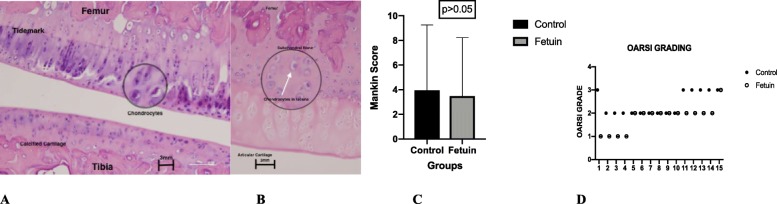
Representative frontal knee articular sections of rat groups, hematoxylin-eosin stain. Mankin Score presented as structure: cellular abnormality: matrix staining. **a** Control Group. 20x magnification. The knee joint is shown, where are labeled the femur and tibia bones, the tidemark and the calcified cartilage. Differentiated chondrocytes are shown in the cycle. (Mankin Score S:C:M 5:3:4). **b** Fetuin Group. 20x magnification. The bone of femur is shown, where the articular cartilage is labeled, the subchondral bone and within the circle, chondrocytes in lacuna are highlighted. (Mankin Score S:C:M 4:3:3). **c** Mankin Score histograms between the two groups at the end of the experiment (*p* > 0.05). **d** Scatter-Plot of the OARSI Grading of the Groups of Control and Fetuin (*p* > 0.05)

We observed the changes in the articular surface in Control and Fetuin group. The results are presented in Table 2, Table 3 and Fig. 2. According to OARSI classification, articular surface discontinuity was noted in both groups (Grade 2), which was more present in the Control group, without any statistical significance between two groups. Also, vertical fissures (Grade 3) were observed in both groups, which was also more present in Control group. Lastly, intact articular surface (Grade 1), with some microscopic cracks into the superficial zone were present in the Group of Fetuin, whereas were completely absent from the Control Group. No subchondral denudation and deformation were observed between both groups. So, the range of scores in Control group was Grade 2 to Grade 3 and the range of scores in Fetuin group was Grade 1 to Grade 3. No statistical difference was signed among the groups. (*p* > 0.05).

**Table 2:** OARSI Classification between groups

OARSI GRADE	Control (n)	Fetuin (*n* = 15)
Grade 1	0	4
Grade 2	9	10
Grade 3	6	1

Results are presented as number (n) of cases in each group. No statistical difference was signed between the groups (*p* > 0.05)

**Table 3:** Histological Findings (Modified Mankin Score in Control and Fetuin Group)

	Control (*n* = 15)	Fetuin (*n* = 15) *P* value (vs control)
Structure	0.15 ± 0.36	0n.s
Cellular abnormalities	1.76 ± 0.70	1.4 ± 0.45 n.s
Matrix Staining	2.1 ± 0.8	2 n.s
Total Score (0–14)	11.88 ± 1.35	10.5 ± 0.8 n.s

Results are presented as the mean ± SD

n.s: non significant

*P* < 0,05 indicates statistical significance

Regarding the Mankin Score, the results are presented in Table 3. No statistically significant difference was found between the two groups.

## Discussion

In the present experimental study, we made an effort to shed light to the possible potential of the intra-articular administration of Fetuin-A to inhibit the progress of post-traumatic secondary osteoarthritis of the knee. Our notable results show that Fetuin-A mildly affects the development of osteoarthritis, on the grounds that only IL-2 levels were significantly lower in the Fetuin Group while CRP levels seemed significantly increased in the Fetuin Group. On the other hand, the levels of the rest of the ILs, BMPs and serum Fetuin-A were not affected by the local administration of Fetuin-A.

Also, histologically, Fetuin Group signed better scores according to the OARSI classification system and the modified Mankin score system, in contrast to Control Group, however no statistical significance was noted.

In 2006, *Hulejova* et al demonstrated the pro-inflammatory condition of OA with increased cytokines and metalloproteinases, as it was described by a clinical trial of 55 patients with severe hip osteoarthritis (Huch et al. 1997). Interleukin-2 (IL-2), is produced by T cells following their activation by antigen. IL-2 induces expression of IL-2 Receptor (IL-2 R) which is expressed on activated lymphocytes and binds IL-2 with low affinity, without signaling domains (Abe et al. 2017). T helper cells (Th) induce the expression of macrophage inflammatory protein (MIP-γ), during the progression of osteoarthritis (OA). The number of CD4 Th cells and the expression of interferon-γ (IFN-γ) are increased during the OA onset, especially after Anterior Crucial Ligament Transection (ACLT). However they seem to decline in later stages of OA. The activation of CD4 T cells leads to increased expression of MIP-1γ which further increases the number of osteoclasts in the joint. It is well stated by the current literature that the amount of CD4 T cells is increased in the peripheral blood in the case of knee osteoarthritis. In our study, the statistically significant difference in the levels of IL-2 was detected in the later stages of the experiment (8 weeks post-operatively), which could be explained by the anti-inflammatory role of the intra-articular administration of Fetuin-A, on the grounds that it enhanced the further decrease in the amounts of IL-2, as CD4 Th cells are mentioned to lower their levels on the later stages of the onset of arthritis.

CRP has also been described as an assessment factor of osteoarthritis. In 2003, *Sturmer* et al associated high sensitive CRP to the progression stage of osteoarthritis in patients with advanced hip and knee osteoarthritis, through a clinical trial of 770 patients (Shen et al. 2011). According to our findings, CRP increased steadily in both groups both in 5 and 8 weeks of the experiment. However, in the fetuin group, in 5 weeks, CRP levels were increased in a statistically significant way in comparison with the control group while in 8 weeks, the levels of CRP were more increased in a statistically significant way in comparison with the baseline levels.

Regarding the rest biomarkers, IL-7 signed increased levels in 5 and 8 weeks in both groups, without any statistical difference. Both IL-2 and IL-7 have been mentioned to lead a pro-inflammatory role in the progress of osteoarthritis, as well as IL-1 and Tumor Necrosis Factor (TNF)-α have been shown to enhance the production of proteolytic enzymes in early OA, leading to fundamental changes in the extracellular matrix which leads to chondrocyte proliferation,stimulated collagen and proteoglycans biosynthesis corresponding to a repair attempt (Hulejová et al. 2007, Zangerle et al. 1992). The anabolic activity of chondrocytes is promoted by growth factors such Insulin-like Growth Factor I (IGF-I). IGF-I along with transforming growth factor (TGF-β) and the related bone morphogenetic protein (BMP) family, are the most significant stimulating agents of chondrocyte production (Goldring and Goldring 2006).

At least 13 proteins are the members of the BMP family (Hedbom and Häuselmann 2002). Some of the BMPs, such as BMP-2,4,7, are outstanding stimulators of articular cartilage proteoglycans production in vitro, while BMP-7 has been found to be effective to overcome the IL-1 promoted downregulation of proteoglycans in human articular chondrocytes in vitro (Hogan 1996). In our case, BMPs levels seemed not to be affected by the administration of Fetuin-A. In control group, BMP-2,4,7 increased slightly in 5 and 8 weeks without any statistical difference, while in Fetuin Group BMP-4,7 decreased slightly in 8 weeks without statistical difference. It could be of great interest to study the BMPs above in longer PTOA protocols, in order to define the proper time periods which could lead to statistical significant results regarding their role in the inflammatory process of PTOA.

Concerning the possible anti-inflammatory role of Fetuin-A on the progress of osteoarthritis previous studies have shown diverse results. *Rittenberg* et al, in 2005, studied the regulated release of regulatory molecules such as fetuin-A by intrarticular injections of BMP7 in clinical trials (Rittenberg et al. 2005, Pritzker et al. 2003). Also, *Wang* et al in 1997 mentioned that Tumor Necrosis Factor (TNF) increase from lipopolysaccharide stimulated macrophages, was attenuated significantly in a study of inflammation also on animal model, where Fetuin-A was administered (Wang et al. 1998). Consequently, the co-administration of Fetuin-A and BMPs is likely to lead a therapeutic intervention for the degenerative bone disease, taking under consideration the anti-inflammatory role of the means above (Brylka et al. 2017). Fetuin-A has been shown to antagonize both the osteogenic and anti-proliferative actions of Tumor Growth Factor (TGF)-β/BMP cytokines in vitro. The administration of Fetuin-A modulates the activity of BMPs and is likely to suppress the immediate cellular immune response of the joint, as it is described in clinical trials with Degenerative Joint Disease (DJD) (Xiao et al. 2013). However, the anti-inflammatory role of Fetuin-A especially combined with the chondro-protective role of BMP-7 is well stated in the current literature (Riordan et al. 2014; Seto et al. 2012a). Moreover, in 2011, *Albilia* et al through a clinical trial of 30 patients requiring joint replacement, described more increased levels of BMP-2,4 and decreased levels of Fetuin-A in patients undergoing total joint replacement with severe joint arthritis (Albilia et al. 2013). In our study, the systematic Fetuin-A levels seemed to be unaffected by the administration of Fetuin-A intra-articularly. In both groups, Fetuin-A levels increased in 5 weeks but in 8 weeks of the experiment, they both decreased slightly, in a non- statistically significant way. Furthermore, in the present study we underlined that Fetuin-A established its anti-inflammatory role histologically, on the grounds that Fetuin Group signed better OARSI and Mankin scores in contrast with the Control Group, yet without statistical significance.

Concerning the histological results, in our study 6 severely affected joints (Grade 3) were observed, all of them in the control group of rats. The rest of the results were both mildly affected or moderately affected according to the OARSI Classification system (Grades 1 and 2). Also, the Modified Mankin Histological-Histochemical Grading System (HHGS) was used as a grading scheme for assessing the osteoarthritis severity (Man and Mologhianu 2014). However, no statistically significant different results among groups were noted. Perhaps more studies and more specific staining is needed, in order to find statistically significant results.

Limitations of this study merit to be mentioned. Firstly, the intra-articular administration of Fetuin-A was performed in a form of bovine fetuin in the rat knees. Secondly, despite the fact that the idea of intra-articular therapy for preventing or slowing the progression of the osteoarthritis of the knee is very topical, the experimental study above did not include an additional group of animals where a placebo could have been administrated intra-articularly after the surgical intervention for the induction of PTOA. Concerning the pro-inflammatory cytokines studied, IL1 and TNFalpha were not included in the present study. Also, the study was focused only on the likely attenuating anti-inflammatory role of Fetuin-A in the knee joint post-traumatic arthritis, so the other properties of Fetuin were not studied, such as its role in joint mineralization and cartilage homeostasis. Additionally, between the existing histological staining that are mentioned above such as Safranin-O, only hematoxylin-eosin was used in the present study, whereas the classification system by Gerwin et al. which was proposed for the OA classification in rats was not included either Gerwin et al. 2010. Moreover, the experimental study did not include immunoassay of the immune cells that are likely to infiltrate the joint during the inflammatory response to PTOA. Lastly, the current experimental model is only carried out on animals, without any clinical trial, so further investigation is needed. However, the present study has significant strength on the grounds that the 30 animals were randomly distributed equally in both groups. Also, the histological specimens were reviewed by a blinded expert. Furthermore, the histological results are accompanied by reduced bias as they are based on the OARSI classification and the modified Mankin classification.

## Conclusions

In conclusion, it is suggested by our results, that the intraarticular administration of Fetuin-A is likely to lead a restrictive role on the attenuation of the progress of post-traumatic secondary osteoarthritis of the knee in rats, on the grounds that only IL-2 was significantly decreased in the Fetuin Group in comparison with the Control Group. Concerning the rest of the biomarkers, the levels of IL-4, IL-7, BMPs and Fetuin-A did not sign any statistically significant difference between groups. An unexpected finding was that CRP levels were significantly increased in the Fetuin Group in contrast with the Control Group. Regarding the histological findings, the Fetuin group signed better scores according to the OARSI Classification Score System and the Modified Mankin Score, yet without any statistical difference. However, current evidence is lacking and additional research is needed regarding the effect of Fetuin-A on the local progression of the secondary osteoarthritis in joints like knee and hip. Hopefully, more light will be shed on mechanisms with which fetuin-A inhibits local inflammation in the case of post-traumatic secondary arthritis.

## Data Availability

The datasets used and/or analysed during the current study are available from the corresponding author on reasonable request. All the data are included in the specific manuscript.

## References

[CR1] Abe S, Nochi H, Ito H (2017). Human articular chondrocytes induce Interleukin-2 nonresponsiveness to allogeneic lymphocytes. Cartilage.

[CR2] Albilia JB, Tenenbaum HC, Clokie CM, Walt DR, Baker GI, Psutka DJ, Backstein D, Peel SA (2013). Serum levels of BMP-2, 4, 7 and AHSG in patients with degenerative joint disease requiring total arthroplasty of the hip and temporomandibular joints. J Orthop Res.

[CR3] Attur M, Krasnokutsky-Samuels S, Samuels J, Abramson SB (2013). Prognostic biomarkers in osteoarthritis. Curr Opin Rheumatol.

[CR4] Baenziger JU, Fiete D (1979). Structure of the complex oligosaccharides of fetuin. J Biol Chem.

[CR5] Bendele AM (2001). Animal models of osteoarthritis. J Musculoskelet Neuronal Interact..

[CR6] Bramlage C, Häupl T, Kaps C, Ungethüm U, Krenn V, Pruss A, Müller G, Strutz F, Burmester G (2006). Decrease in expression of bone morphogenetic proteins 4 and 5 in synovial tissue of patients with osteoarthritis and rheumatoid arthritis. Arthritis Res Ther.

[CR7] Brylka LJ, KoÈppert S, Babler A, Kratz B, Denecke B, Yorgan TA (2017). Post-weaning epiphysiolysis causes distal femur dysplasia and foreshortened hindlimbs in fetuin-A-deficient mice. PLoS One.

[CR8] Chambers MG, Bayliss MT, Mason RM (1997). Chondrocyte cytokine and growth factor expression in murine osteoarthritis. Osteoarthritis Cartilage..

[CR9] Chubinskaya S, Hurtig M, Rueger DC (2007). OP-1/BMP-7 in cartilage repair. Int Orthop.

[CR10] Gerwin N, Bendele A, Glasson S, Carlson C (2010). The OARSI histopathology initiative e recommendations for histological assessments of osteoarthritis in the rat. Osteoarthr Cartil.

[CR11] Glasson SS, Blanchet TJ, Morris EA (2007). The surgical destabilization of the medial meniscus (DMM) model of osteoarthritis in the 129/SvEv mouse. Osteoarthr Cartil.

[CR12] Goldring SR, Goldring MB (2006). Clinical aspects, pathology and pathophysiology of osteoarthritis. J Musculoskelet Neuronal Interact.

[CR13] Hedbom E, Häuselmann E (2002). Molecular aspects of pathogenesis in osteoarthritis: the role of inflammation. Cell Mol Life Sci.

[CR14] Hogan BL (1996). Bone morphogenetic proteins: multifunctional regulators of vertebrate development. Genes Dev.

[CR15] Huch K, Wilbrink B, Flechtenmacher J, Koepp HE, Aydelotte MB, Sampath TK (1997). Effects of recombinant human osteogenic protein 1 on the production of proteoglycan, prostaglandin E2, and interleukin-1 receptor antagonist by human articular chondrocytes cultured in the presence of interleukin-1beta. Arthritis Rheum.

[CR16] Hulejová H, Baresová V, Klézl Z, Polanská M, Adam M, Senolt L (2007). Increased level of cytokines and matrix metalloproteinases in osteoarthritic subchondral bone. Cytokine.

[CR17] Kraus V.B., Burnett B., Coindreau J., Cottrell S., Eyre D., Gendreau M., Gardiner J., Garnero P., Hardin J., Henrotin Y., Heinegård D., Ko A., Lohmander L.S., Matthews G., Menetski J., Moskowitz R., Persiani S., Poole A.R., Rousseau J.-C., Todman M. (2011). Application of biomarkers in the development of drugs intended for the treatment of osteoarthritis. Osteoarthritis and Cartilage.

[CR18] Kuyinu E, Narayanan G, Nair L, Laurencin C (2016). Animal models of osteoarthritis: classification, update, and measurement of outcomes. J Orthop Surg Res.

[CR19] Lampropoulou-Adamidou K, Lelovas P, Karadimas EV, Liakou C, Triantafillopoulos IK, Dontas I (2014). Useful animal models for the research of osteoarthritis. Eur J Orthop Surg Traumatol.

[CR20] Little CB, Hunter DJ (2013). Post-traumatic osteoarthritis: from mouse models to clinical trials. Nat Rev Rheumatol.

[CR21] Liu Y, Hou R, Yin R, Yin W (2015). Correlation of bone morphogenetic Protein-2 levels in serum and synovial fluid with disease severity of knee osteoarthritis. Med Sci Monit.

[CR22] Man G, Mologhianu G (2014). Osteoarthritis pathogenesis—a complex process that involves the entire joint. J Med Life.

[CR23] Mankin H, Dorfman H, Lippiello L, Zarins A (1971). Biochemical and metabolic abnormalities in articular cartilage from osteoarthritic human hips, II: correlation of morphology with biochemical and metabolic data. J Bone Joint Surg Am.

[CR24] McNulty M, Loeser R, Davey C, Callahan M, Ferguson C, Carlson C (2011). A Comprehensive Histological Assessment of Osteoarthritis Lesions in Mice. Cartilage.

[CR25] Pappa E, Perrea DS, Pneumaticos S, Nikolaou VS (2017). Role of fetuin a in the diagnosis and treatment of joint arthritis. World J Orthop.

[CR26] PEDERSEN KAI O. (1944). Fetuin, a New Globulin Isolated from Serum. Nature.

[CR27] Ponchel P, Burska A, Hensor E, Raja R, Campbell M, Emery P, Conaghan P (2015). Changes in peripheral blood immune cell composition in osteoarthritis. Osteoarthr Cartil.

[CR28] Pongratz G, Anthofer J, Melzer M, Anders S, Grässel S, Straub R (2014). IL-7 receptor α expressing B cells act proinflammatory in collagen-induced arthritis and are inhibited by sympathetic neurotransmitters. Ann Rheum Dis.

[CR29] Pritzker KPH. Pathology of osteoarthritis. In: Brandt KD, Doherty M, Lohmander LS, (2003) Eds. Osteoarthritis. 2^nd^ edn. Oxford University Press 49e58, Oxford

[CR30] Pritzker K.P.H., Gay S., Jimenez S.A., Ostergaard K., Pelletier J.-P., Revell P.A., Salter D., van den Berg W.B. (2006). Osteoarthritis cartilage histopathology: grading and staging. Osteoarthritis and Cartilage.

[CR31] Pritzker KPH, Laverty SP, Mendes MG (2003). Histopathologic grading of experimental osteoarthritis. Osteoarthr Cartil.

[CR32] Riordan EA, Little C, Hunter D (2014). Pathogenesis of post-traumatic OA with a view to intervention. Best Pract Res Clin Rheumatol.

[CR33] Rittenberg B, Partridge E, Baker G, Clokie C, Zohar R, Dennis JW, Tenenbaum HC (2005). Regulation of BMP-induced ectopic bone formation by Ahsg. J Orthop Res.

[CR34] Seto Jong, Busse Björn, Gupta Himadri S., Schäfer Cora, Krauss Stefanie, Dunlop John W. C., Masic Admir, Kerschnitzki Michael, Zaslansky Paul, Boesecke Peter, Catalá-Lehnen Philip, Schinke Thorsten, Fratzl Peter, Jahnen-Dechent Willi (2012). Accelerated Growth Plate Mineralization and Foreshortened Proximal Limb Bones in Fetuin-A Knockout Mice. PLoS ONE.

[CR35] Shen Y, Wu C, Jou I, Lee C, Juan H, Lee P, Chen S, Hsieh L (2011). T helper cells promote disease progression of osteoarthritis by inducing macrophage inflammatory protein-1g. Osteoarthr Cartil.

[CR36] Sluijs JA, Geesink RGT, van der Linden AJ, Bulstra SK, Kuyer R, Drukker J (1992). The reliability of the Mankin score for osteoarthritis. J Orthop Res.

[CR37] Sturmer T, Brenner H, Koenig W, Gunther K (2004). Severity and extent of osteoarthritis and low grade systemic inflammation as assessed by high sensitivity C reactive protein. Ann Rheum Dis.

[CR38] Wang H, Li W, Zhu S, Li J, Ward MF, Huang Y, Yang H, Tracey KJ, Wang P, Sama AE (2010). Fetuin protects mice against lethal sepsis by modulating bacterial endotoxin induced hmgb1 release and autophagy. Shock.

[CR39] Wang H, Sama AE (2012). Anti-inflammatory role of fetuin-a in injury and infection. Curr Mol Med.

[CR40] Wang H, Zhang M, Bianchi M, Sherry B, Sama A, Tracey KJ (1998). Fetuin (alpha2-HS-glycoprotein) opsonizes cationic macrophagedeactivating molecules. Proc Natl Acad Sci U S A.

[CR41] Wang H, Zhang M, Soda K, Sama A, Tracey KJ (1997). Fetuin protects the fetus from TNF. Lancet.

[CR42] Xiao J, Wang XR, Hu KZ, Li MQ, Chen JW, Ma T, Li ZC (2013). Serum fetuin-A levels are inversely associated with clinical severity in patients with primary knee osteoarthritis. Biomarkers.

[CR43] Zangerle PF, De Groote D, Lopez M, Meuleman RJ, Vrindts Y, Fauchet F, Dehart I, Jadoul M, Radoux D, Franchimont P (1992). Direct stimulation of cytokines (IL-1 beta, TNF-alpha, IL-6, IL-2, IFN-gamma and GM-CSF) in whole blood: II. Application to rheumatoid arthritis and osteoarthritis. Cytokine.

