# Correction to: The role of intra-articular administration of Fetuin-A in post-traumatic knee osteoarthritis: an experimental study in a rat model

**DOI:** 10.1186/s40634-019-0201-9

**Published:** 2019-07-11

**Authors:** Eleni Pappa, Savvas Papadopoulos, Laskarina-Maria Korou, Despina N. Perrea, Spiridon Pneumaticos, Vasileios S. Nikolaou

**Affiliations:** 1 “KAT” General Hospital of Athens, Nikis 2, 14561 Kifisia, Greece; 2 Department of Pathology, “Hygeia” General Hospital of Athens, Athens, Greece; 3 Laboratory of Experimental Surgery and Research “N.S. Christeas”, Athens Medical School, Athens, Greece; 4 3rd Department of Orthopaedics, KAT Hospital, National and Kapodistrian University of Athens, School of Medicine, Athens, Greece; 5 2nd Department of Orthopaedics, Agia Olga Hospital, National and Kapodistrian University of Athens, School of Medicine, Athens, Greece

**Correction to: J Exp Orthop (2019) 6:25**

**https://doi.org/10.1186/s40634-019-0194-4**

Following publication of the original article (Pappa et al. 2019), the authors opted to correct the middle initial of co-author Despina N. Perrea from S to N. The original article has been corrected.
